# Interaction of basolateral amygdala, ventral hippocampus and medial prefrontal cortex regulates the consolidation and extinction of social fear

**DOI:** 10.1186/s12993-018-0139-6

**Published:** 2018-03-19

**Authors:** Chu-Chu Qi, Qing-Jun Wang, Xue-zhu Ma, Hai-Chao Chen, Li-Ping Gao, Jie Yin, Yu-Hong Jing

**Affiliations:** 1 Institute of Anatomy and Histology & Embryology, Neuroscience, School of Basic Medical Sciences, Lanzhou University, No. 199 of Donggang West Road, Lanzhou, 730000 Gansu People’s Republic of China; 2 Key Laboratory of Preclinical Study for New Drugs of Gansu Province, Lanzhou University, No. 199 of Donggang West Road, Lanzhou, 730000 Gansu People’s Republic of China; 3 Institute of Biochemistry and Molecular Biology, School of Basic Medical Sciences, Lanzhou University, No. 199 of Donggang West Road, Lanzhou, 730000 Gansu People’s Republic of China

**Keywords:** Social defeat, Information extinction, Basolateral amygdala, Ventral hippocampus, Medial prefrontal cortex

## Abstract

**Background:**

Following a social defeat, the balanced establishment and extinction of aversive information is a beneficial strategy for individual survival. Abnormal establishment or extinction is implicated in the development of mental disorders. This study investigated the time course of the establishment and extinction of aversive information from acute social defeat and the temporal responsiveness of the basolateral amygdala (BLA), ventral hippocampus (vHIP) and medial prefrontal cortex (mPFC) in this process.

**Methods:**

Mouse models of acute social defeat were established by using the resident–intruder paradigm. To evaluate the engram of social defeat, the intruder mice were placed into the novel context at designated time to test the social behavior. Furthermore, responses of BLA, vHIP and mPFC were investigated by analyzing the expression of immediate early genes, such as zif268, arc, and c-fos.

**Results:**

The results showed after an aggressive attack, aversive memory was maintained for approximately 7 days before gradually diminishing. The establishment and maintenance of aversive stimulation were consistently accompanied by BLA activity. By contrast, vHIP and mPFC response was inhibited from this process. Additionally, injecting muscimol (Mus), a GABA receptor agonist, into the BLA alleviated the freezing behavior and social fear and avoidance. Simultaneously, Mus treatment decreased the zif268 and arc expression in BLA, but it increased their expression in vHIP.

**Conclusion:**

Our data support and extend earlier findings that implicate BLA, vHIP and mPFC in social defeat. The time courses of the establishment and extinction of social defeat are particularly consistent with the contrasting BLA and vHIP responses involved in this process.
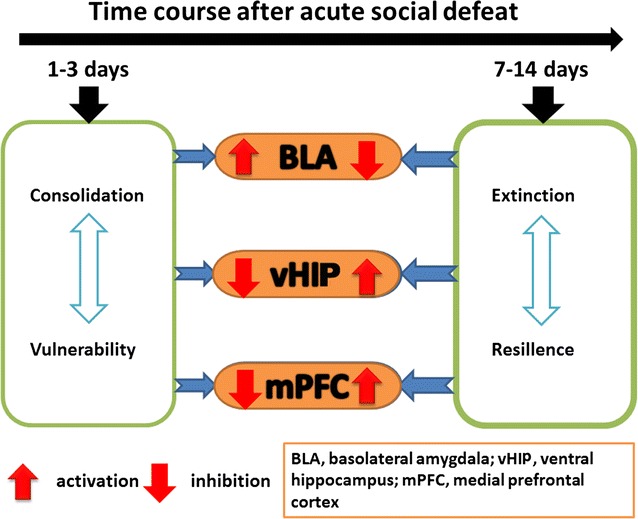

**Electronic supplementary material:**

The online version of this article (10.1186/s12993-018-0139-6) contains supplementary material, which is available to authorized users.

## Background

World Health Organization has identified mood disorders as a major cause of mortality, morbidity and disability worldwide [1, 2]. Social defeat is a critical phenotype of mood disorders, including severe depression, anxiety, bipolar disorder, autism and schizophrenia. Also social defeat is manifested by symptoms such as social fear, social avoidance and social withdrawal [3–5]. Overcoming social defeat is the primary determinant of the efficacy of drugs and therapeutic methods on mental disorders. However, the etiology and mechanisms of social defeat remain poorly understood.

Social defeat is induced by physical and psychological stresses, and implicates the cerebral cortex and multiple brain regions of the limbic system [6–8]. Among these regions, the basolateral amygdala (BLA) establishes and consolidates conditioned fears and harmful stimulations. The establishment of conditioned defeat is impaired by infusing the BLA with anisomycin to inhibit protein synthesis. The consolidation of conditioned defeat is enhanced by overexpressing the cyclic adenosine monophosphate (cAMP) response element binding protein in the BLA through a viral vector [9–11]. Therefore, fear consolidation involves a new type of protein synthesis and the formation of neural microcircuit in the BLA. The medial prefrontal cortex (mPFC) is associated with the motivation and the integration in the development of depression and autism. On the one hand, stress stimulation inhibits the mPFC. On the other hand, mPFC activity caused by optogenetics or deep brain stimulation alleviates the symptoms of depression, and which partially contributes to fear extinction through unclear mechanisms [12, 13]. The hippocampus plays pivotal roles in regulating social behavior. Significant evidence indicates that the dorsal and ventral hippocampus (vHIP) play different roles in processing fear and harmful information from context and conditional stimulation [14]. The bidirectional neural circuit between the vHIP and the hypothalamus integrates harmful information and then determines the vulnerability or resilience toward stress stimulation [15].

The severity of social defeat is determined by the strength and duration of the stimuli. Although different parameters have been considered in establishing rodent models of social defeat, the key criteria are based on common symptoms, including social avoidance, social fear and social withdrawal [16–18]. Social defeat is established in adult rodent through one or more harmful stimulation, followed by gradual recovery through stress removal. The appropriate social strategy is selected according to past experience and learning ability, involving the acquisition, consolidation, retrieval and extinction of social information under specific conditions. Nevertheless, the duration of social defeat is determined by different behavioral paradigms. The changes in the behavior and responses of brain regions are difficult to correlate with one another. Literature has focused on the formation of social defeat and its mechanisms. Few studies have investigated the process of social defeat from establishment to extinction. Our work aims to explore the time characteristics of the consolidation and extinction of acute social defeat and the simultaneous responses of BLA, vHIP, and mPFC. We utilized the resident–intruder paradigm to establish an animal model of acute social defeat. The duration of the consolidation and extinction of social defeat were evaluated. The temporal features of the responses of BLA, vHIP, and mPFC to acute social defeat were also investigated by quantifying the expression of immediate early genes (IEGs), namely, zif268, arc, and c-fos, which are indicators of neural activity.

## Methods

### Materials

Muscimol (Mus, cat. G019–5 Mg) and rabbit polyclonal anti-c-fos (cat. HPA018531) were obtained from Sigma (St. Louis, MO, USA). Mouse monoclonal anti-c-fos (cat. Sc8047) and GAPDH (cat. Sc-69778) were obtained from Santa Cruz (Santa Cruz, CA, USA). Rabbit polyclonal anti-parvalbumin antibody (cat. bs-1299R) was purchased from Bioss (Bioss technology company, Beijing, China). Based on the sequences retrieved from a gene bank, Mouse zif268 (accession: NM007913) and arc (accession: NM001276684) primers were designed and produced by Takara Biotechnological Company (Takara Biotech, Co., Ltd., Dalian, China). Kits (cat. 9108) for total RNA extraction were purchased from Takara Biotechnological Company (Takara Biotech, Co., Ltd., Dalian, China). SYBR Green PCR Master Kits (cat. A6001) were purchased from promega (Promega Corporation, USA).

### Animals

Male C57BL/6 mice weighing approximately 20–22 g and male Kunming (KM) mice weighing approximately 35–40 g were obtained from the Experimental Center of Lanzhou University. Animals were reared in a clean house under a 12-h light/dark cycle, temperature of 22–25 °C, and humidity of 50–60%. Mice were fed with regular diet and purified water ad libtum. All experiments were conducted in accordance with the guidelines of the Policies Governing the Use of Live Vertebrate Animals of the Lanzhou University.

### Social defeat

One KM mouse raised alone in a cage for 2 weeks was designated as the resident. The C57BL/6 mice were randomly assigned to a control without the social attack (n = 12) and social defeat with the social attack according to the previous methods [19]. In brief, a C57BL/6 mouse was introduced as the intruder. For 10 min, the C57BL/6 mouse was exposed to the cage with the KM mouse. The aggressive attack was stopped using a perforated plastic barrier, which allows for continued visual, auditory and olfactory contact. After 6 h, the barrier was removed and physical contact was allowed for 10 min. After the second attack, the C57BL/6 mouse was placed back in its home cage. Cumulated frequency of attack is about 40 times per mouse. Mice in social defeat were divided into subgroups on the basis of the designated time at which the animals were sacrificed after social aggression (1, 3, 7, and 15 days; n = 12 per subgroup).

### Stereotactic injections

C57BL/6 mice were anesthetized with isoflurane after the completion of social aggression. Mus (40 ng/0.25 µl) or an equal volume of saline was bilaterally injected into the BLA (bregma coordinates: AP − 1.7 mm; ML ± 3.3 mm; DV − 3.8 mm from the brain surface). After 3 days, mice were sacrificed following behavioral examination.

### Open field test

While exploring a 50 cm × 50 cm open-field arena, the behavior of the mice was assessed during a 5-min test. Locomotor activity and freezing time were measured by a video-tracking system (TM-vision, Chengdu Techman Software Co., Ltd., Chengdu, China).

### Social interaction

Social fear and avoidance were assessed using the modified open-field system [20]. Briefly, two 9 cm × 9 cm mesh enclosures were placed at the opposite sides of the open field. One enclosure contained a novel KM mouse as the social target, and another enclosure did not contain a mouse as the social un-target. The experimental mouse was allowed to freely explore a 50 cm × 50 cm arena containing the target and un-target enclosures. The time spent on the social interaction zone (14 cm × 26 cm) surrounding the target enclosure and the reference zones (14 cm × 26 cm) surrounding the un-target enclosure was measured using a video tracking system (TM-vision, Chengdu Techman Software Co., Ltd., Chengdu, China).

### Sucrose preference

This test consisted of a 2-bottle procedure in which mice were given the choice between consuming water or a 1% sucrose solution according to previously described method with modification [21]. In brief, animals were singly housed in a cage that had two drinking bottles. One of the bottles had water, while the other bottle had a 1% sucrose solution. Water and sucrose consumption was measured the following day (8:00 A.M.). The position of the sucrose bottle was counterbalanced (left versus right) across the different cages to control for potential side-preference bias.

### Marble-burying test

The marble-burying test was performed according to previously described methods with minor modifications [22]. Briefly, the apparatus consisted of a plastic box (15 × 10 × 10 cm) and 9 clean glass marbles (10 mm diameter) that were evenly spaced (4 cm apart) on sawdust (2 cm deep). The number of marbles that were at least 2/3 buried after 5 min was recorded.

### Histology

Following behavioral testing, mice were returned to their home cages. After 2 h, mice were deeply anesthetized with isoflurane and transcardially perfused with 20 ml of heparinized saline solution and then with 30 ml of 4% paraformaldehyde in 0.1 M phosphate buffer (PB) at pH 7.4 and 4 °C. Brains were harvested and stored in the same fixative for 90 min at 4 °C followed by at least 24 h of immersion in 20% sucrose solution in 0.1 M PB with 4% paraformaldehyde. Coronal Sects. (30 µm thickness) were obtained using a cryostat microtome. Sections containing the BLA were mounted onto glass slides and counterstained with Cresyl Violet to precisely locate the microinjection site under light microscopy. The remaining sections were used for immunohistochemistry.

### Immunohistochemistry

c-Fos immunolabelling was performed using a rabbit polyclonal c-Fos antibody. In brief, sections were quenched in 0.3% hydrogen peroxide (H_2_O_2_) for 20 min to remove endogenous peroxidase activity. After serial washing in 0.01 M phosphate- buffered saline (PBS), sections were incubated in phosphate-buffered saline containing rabbit anti c-fos antibody (1:200), 0.3% Triton X, and 0.1% bovine serum albumin (BSA) for 24 h at 4 °C. The incubated sections were then washed and incubated for 90 min in biotinylated goat-anti-rabbit antisera (1:200), followed by washing in 0.01 M PBS. Sections were then incubated in Strep-avidin–biotin–peroxidase complex (ABC) (1:200) for 90 min and then immersed in 0.02% 3,3-diaminobenzidine (DAB) containing 0.01% H_2_O_2_ in 0.01 M PBS until a brown reaction product developed.

### Quantification of immunohistochemical staining

Photomicrographs were taken with an Olympus microscope BX3 and Olympus DP73 digital camera. Regions of interest were defined in accordance with the mouse brain atlas based on specific landmarks that comprise cellular groups. The anterior–posterior (AP) level from the bregma of the analyzed regions was as follows: BLA (AP: − 0.6 mm to − 0.26 mm), vHIP (AP: -2.8 mm to -3.8 mm) and mPFC (AP: 2.34 mm–1.34 mm). c-Fos immunoreactive profiles were captured from a fixed area under 200 × magnification in at least 3 sections per region per mouse and quantified using ImageJ software (NIH, Bethseda, MD). Given that c-Fos expression levels did not differ between the left and right sides regions in response to any of the experimental procedures, counts were averaged to yield the mean number of c-fos-positive profiles per region per animal.

### RNA extraction and quantitative real-time PCR

Following behavioral testing, mice were returned to their home cages. After 2 h, mice were deeply anesthetized with isoflurane and sacrificed. The mouse skull was opened and whole brain was removed. Fresh tissues of BLA, vHIP and mPFC were isolated from the corresponding coronal sections under the stereomicroscope. Total RNA was extracted from the BLA, vHIP or mPFC using RNAiso plus reagent (Takara Biotech, Co., Ltd., Dalian, China) in accordance with the manufacturer’s instructions. DNA contamination was removed with RNase-free DNase. cDNA was synthesized from 1 µg of RNA with M-MuLV reverse transcriptase and random hexamer following the manufacturer’s instructions (Fermentas, Burlington, Canada). Quantitative real-time PCR (Q-RT-PCR) was performed using PIKoREAl96 detector (Thermo Scientific, USA). The mRNA levels of zif268 and arc in triplicate samples of reverse-transcribed cDNA were checked with SYBR Green PCR Master Kit (Promega Corporation, USA) in accordance with the manufacturer’s instructions. The primers for mouse zif268 were 5′-CGAACAACCCTATGAGCACCTG-3′ (forward) and 5′-GGCTGGGATAACTCGTCTCCAC-3′ (reverse). The primers for mouse arc were 5′-GCCAAACCCAATGTGATCCTG-3′ (forward) and 5′-CTGCTTGGACACTTCGGTCAAC-3′ (reverse). The mouse gapdh primers were 5′-GCGAGACCCCACTAACATCAA-3′ (forward) and 5′-GTGGTTCACACCCATCACAAA-3′ (reverse). The assays were initiated for 5 min at 95 °C, 40 cycles of 15 s at 94 °C, and 1 min at 60 °C. The threshold cycles of the target gene and gapdh were calculated. The amplification of zif268 and arc cDNA was normalized to the expression of gapdh. The relative mRNA expression levels of zif268 and arc were calculated using the 2^ΔCT^ method.

### Protein extraction and Western blot analysis

Following behavioral testing, mice were returned to their home cages. After 2 h, mice were deeply anesthetized with isoflurane and sacrificed. The mouse skull was opened and whole brain was removed. Fresh tissues of BLA, vHIP and mPFC were isolated from the corresponding coronal sections under the stereomicroscope. Total proteins were extracted from BLA, vHIP or mPFC samples using RIPA buffer that contained protease inhibitors. The extracted proteins (50 g) were fractionated on 10% sodium dodecyl sulfate polyacrylamide gel, and then transferred onto polyvinylidene fluoride membranes. The membranes were blotted with anti-c-Fos (1:1000), anti-GAPDH (1:5000), and horseradish peroxidase-conjugated second antibodies (1:5000). Immunoreactive protein bands were visualized by enhanced chemiluminescence using the Bioanalytical imaging system (Azure Biosystems, INC, USA).

### Statistical analysis

The data were expressed the mean ± SEM. Statistical analysis was performed using SPSS statistical program, version 17.0. The difference between the two groups was analyzed by Student’s t test, whereas that among three or more groups was analyzed by one-way analysis of variance with least significant difference test. A difference with p < 0.05 was considered statistically significant.

## Results

### Behavioral changes after acute aggressive stimulation

**Fig. 1:**
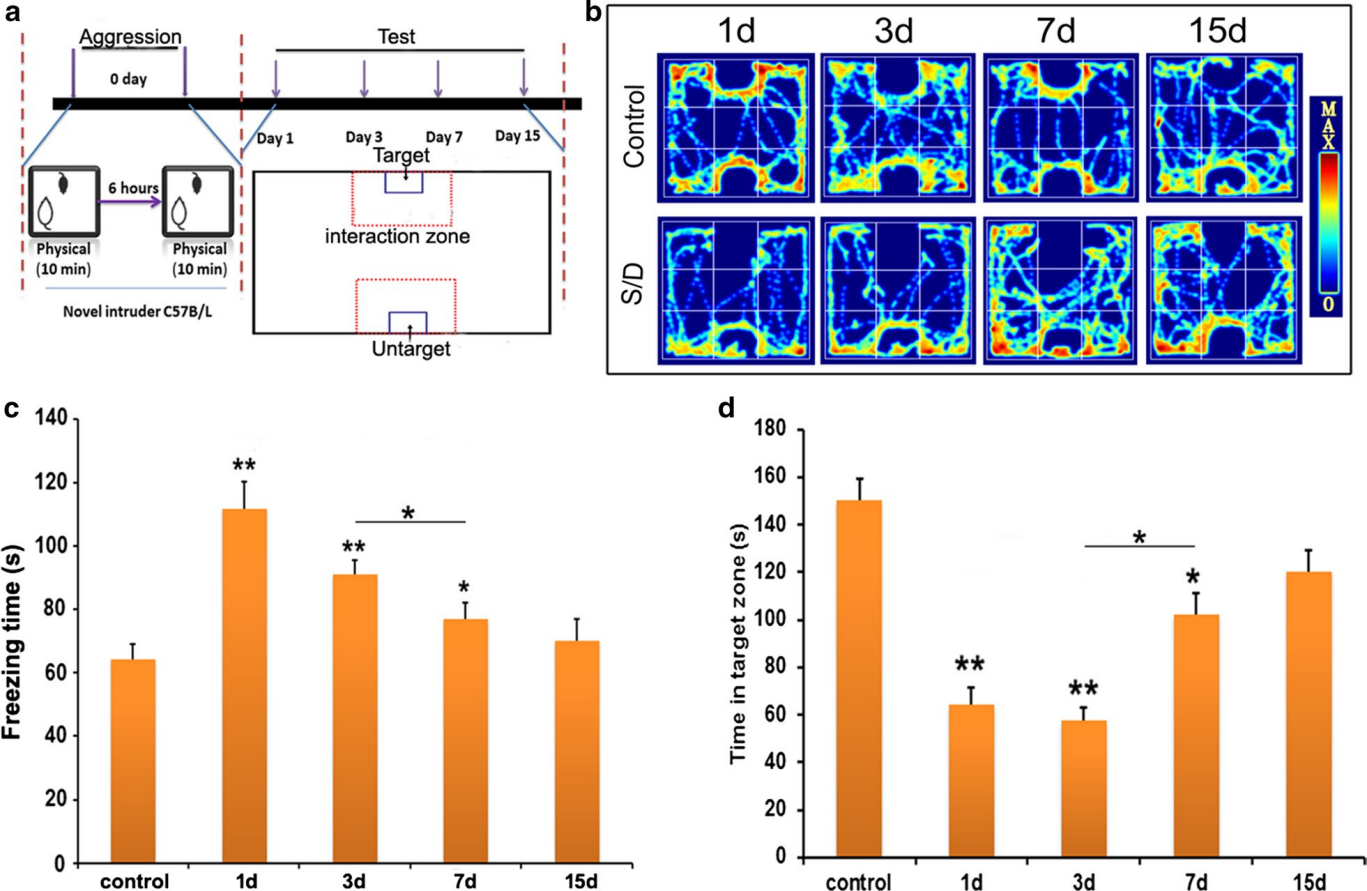
Behavioral changes after acute social aggressive stimulation. **a** Diagram of social aggression and social behavioral test. Social aggression is based on the resident (KM mouse)—intruder (C57B6/L mouse) paradigm. **b** Representative images of the track during social interaction. **c** Freezing time in the open field. On days 1 and 3 after stimulation, the freezing time of intruder mice was longer than that of control mice. On day 7 after stimulation, the freezing time of intruder mice was shorter than that at day 1 after stimulation but remained longer than that of control mice, one way ANOVA with Tukey’s test was used, F = 4.46, *p < 0.05, **p < 0.01 compared with control, n = 12. **d** Time spent in the social interaction zones. On days 1 and 3 after stimulation, intruder mice spent less time in the interaction zone than the control mice. On day 7 after stimulation, intruder mice spent more time in the interaction zone than at days 1 and 3 after stimulation but still spent less time in the interaction zone than control mice, one way ANOVA with Tukey’s test was used, F = 8.98, *p < 0.05, **p < 0.01 compared with control, n = 12

Spontaneous exploration behavior was examined using the open-field test. As shown in Fig. 1c, the freezing time of the intruder mice increased dramatically at days 1 and 3 after stimulation compared with the control mice (F = 4.46, p < 0.01). At day 7 after stimulation, the freezing time of the intruder mice was shorter than that at day 1 after stimulation (F = 4.46, p < 0.05) but was longer than that of the control mice (F = 4.46, p < 0.05). The freezing time of the intruder mice at day 15 after social stimulation was the same as that of the control mice (F = 4.46, p > 0.05). The above data suggests that aversive information from acute stimulation is maintained at least for 7 days before gradually diminishing. The intruder mice recovered their exploratory behavior at 15 days after social defeat. The time spent on the social interaction zone is shown in Fig. 1b, d. At day 1 and day 3 after social aggression, intruder mice spent less time on the interaction zone compared with the control mice (F = 8.98, p < 0.01). On day 7 after stimulation, intruder mice spent more time on the interaction zone than on days 1 and 3 (F = 8.98, p < 0.05) but still spent less on the interaction zone than control mice (F = 8.98, p < 0.05). On day 15 after stimulation, the intruder mice spent the same time on the interaction zone as control mice (F = 8.98, p > 0.05). These data suggest that information for social defeat stress from acute social aggression is maintained for 7 days before diminishing.

### Response of the BLA to social defeat stress

**Fig. 2:**
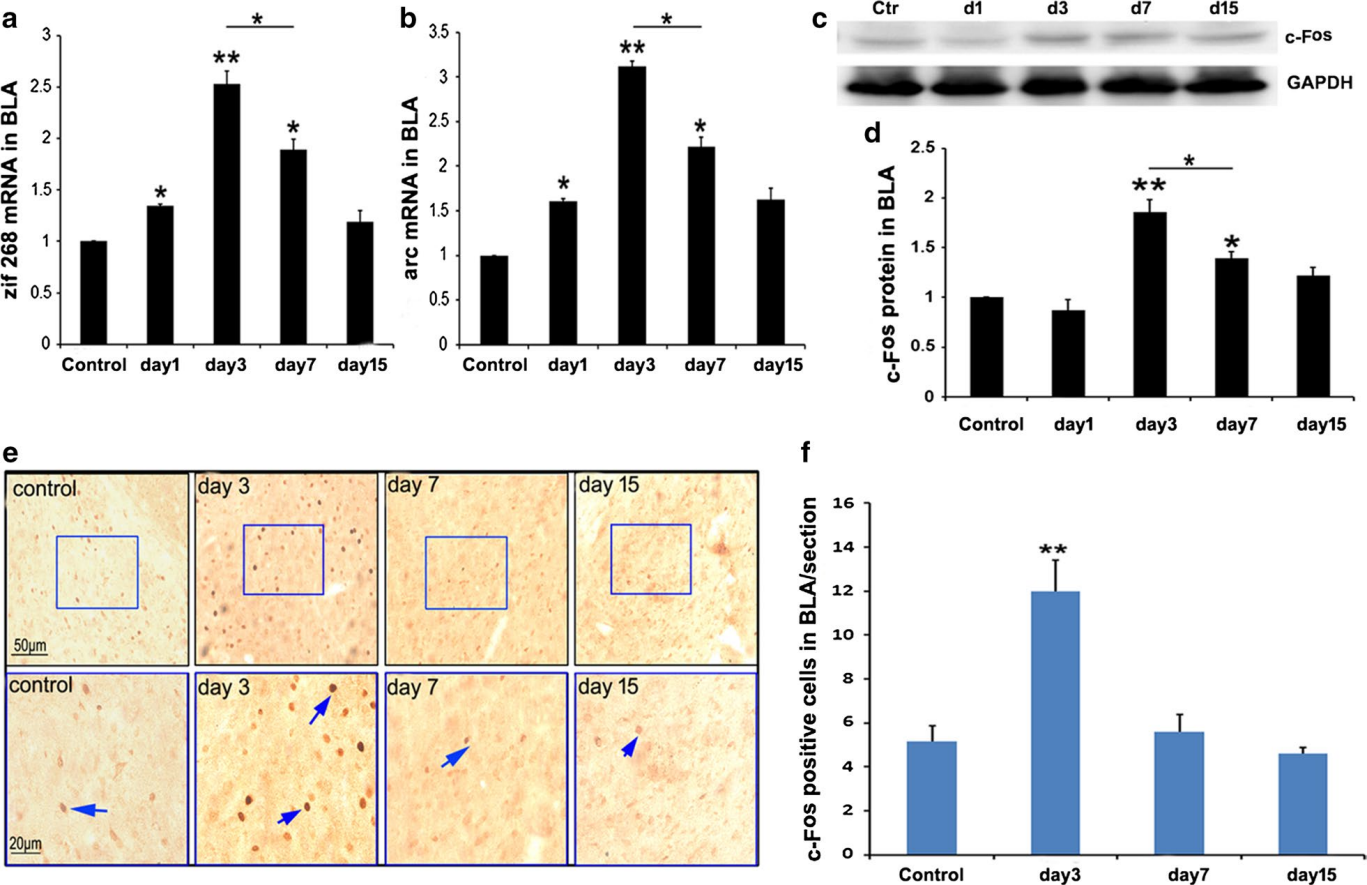
Response of BLA to social defeat stress. **a** mRNA levels of zif 268 and arc in BLA tissues gradually increased at day 1 after stimulation, peaked at day 3 after stimulation, and decreased at day 7 after stimulation compared with control mice, one way ANOVA with Tukey’s test was used, F = 21.5, *p < 0.05, **p < 0.01 compared with the control, n = 8. **b** mRNA levels of arc in BLA tissues gradually increased at day 1 after stimulation, peaked at day 3 after stimulation, and decreased at day 7 after stimulation compared with control mice, one way ANOVA with Tukey’s test was used, F = 26.7, *p < 0.05, **p < 0.01 compared with the control, n = 8. **c** Representative image of the Western blot of c-Fos. **d** Statistical analysis of c-Fos expression, one way ANOVA with Tukey’s test was used, F = 5.6, *p < 0.05, **p < 0.01 compared with the control, n = 8. **e** Representative images of c-Fos staining in BLA. Squares in the upper images indicate areas that are shown magnified in the bottom images. Blue arrows indicate c-Fos-positive cells. **f** Cell count in the BLA, one way ANOVA with Tukey’s test was used, F = 16.357, **p < 0.01 compared with the control, n = 4

Immediate early genes, including zif268, arc and c-fos were used as indices for the evaluation of neural activity. We detected the expression levels of these genes through Q-RT-PCR, Western blotting, and immunohistochemistry. First, we analyzed the mRNA levels of zif268 and arc by using Q-RT-PCR. As shown in Fig. 2a (F = 21.5), b (F = 26.7), the mRNA levels of zif268 and arc in intruder mice gradually increased at day 1, peaked at day 3, decreased at day 7, and were the same as those in control mice at day 15 after stimulation. Second, we examined c-Fos protein levels by using Western blotting. As shown in Fig. 2c, d (F = 5.6), c-Fos expression in intruder mice dramatically increased at day 3 and then slightly decreased at day 7. c-Fos protein levels in intruder mice on days 3 and 7 after stimulation were higher than those in control mice. c-Fos positive cells were labeled and counted as described in “Methods” section. As shown in Fig. 2e, f (F = 16.357), the number of c-Fos positive cells dramatically increased at day 3 after stimulation and then decreased. The above data suggests that aggressive stimulation facilitates BLA activation at the beginning of the stimulus (from day 1 to day 7). This response then diminished during the later stages of the stimulus (from day 7 to day 15).

### Response of vHIP to social defeat stress

**Fig. 3:**
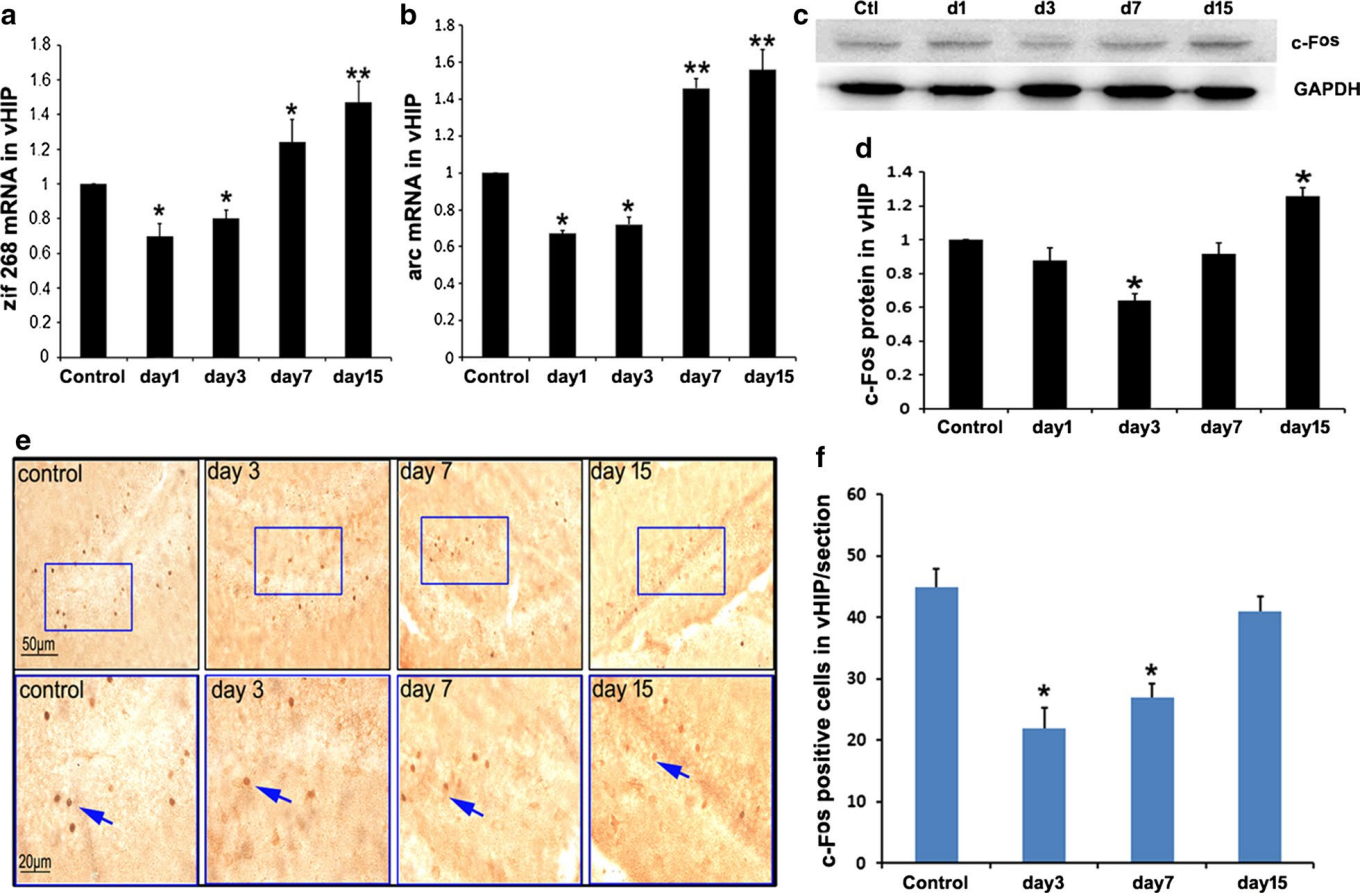
Response of vHIP to social defeat stress. **a** mRNA levels of zif268 in vHIP tissues from intruder mice decreased at days 1 and 3 after stimulation and increased at days 7 and 15 after stimulation compared with those in the control mice, one way ANOVA with Tukey’s test was used, F = 22.233, p < 0.05, **p < 0.01 compared with the control, n = 8. **b** mRNA levels of arc in vHIP tissues from intruder mice decreased at days 1 and 3 after stimulation and increased at days 7 and 15 after stimulation compared with those in the control mice, one way ANOVA with Tukey’s test was used, F = 23.655, p < 0.05, ** p < 0.01 compared with the control, n = 8. **c** Representative Western blot image of c-Fos. **d** Statistical analysis of c-Fos expression, one way ANOVA with Tukey’s test was used, F = 4.73, *p < 0.05, compared with the control, n = 8. **e** The representative images of c-Fos staining in vHIP. Squares in the upper images indicate areas that are shown magnified in the bottom images. Blue arrows indicate c-Fos-positive cells. **f** Cell counts in the vHIP, one way ANOVA with Tukey’s test was used, F = 5.287, *p < 0.05 compared with control, n = 4

zif268, arc mRNA were analyzed by Q-RT-PCR. As shown in Fig. 3a (F = 22.233), b (F = 23.655), the levels of these genes decreased on days 1 and 3 after stimulation and increased at days 7 and 15 after stimulation. We also analyzed the expression of c-Fos protein. Similar to those of zif268 and arc, the expression of c-Fos protein decreased at day 3 and increased at day 15 after stimulation (Fig. 3c, d, F = 4.73). c-Fos-positive cells were labeled and counted as described in "Methods" section. As shown in Fig. 3e, f (F = 5.287), the number of c-Fos-positive cells in intruder mice decreased significantly at days 3 and 7 after stimulation compared with those in the control mice, and then increased to the same levels as those in the control mice at day 15 after stimulation. These results suggest that vHIP is inhibited by aggressive stimulation at the beginning of aggression (from day 1 to day 3), and then gradually recovered (from day 7 to day 15).

### Response of mPFC to social defeat stress

**Fig. 4:**
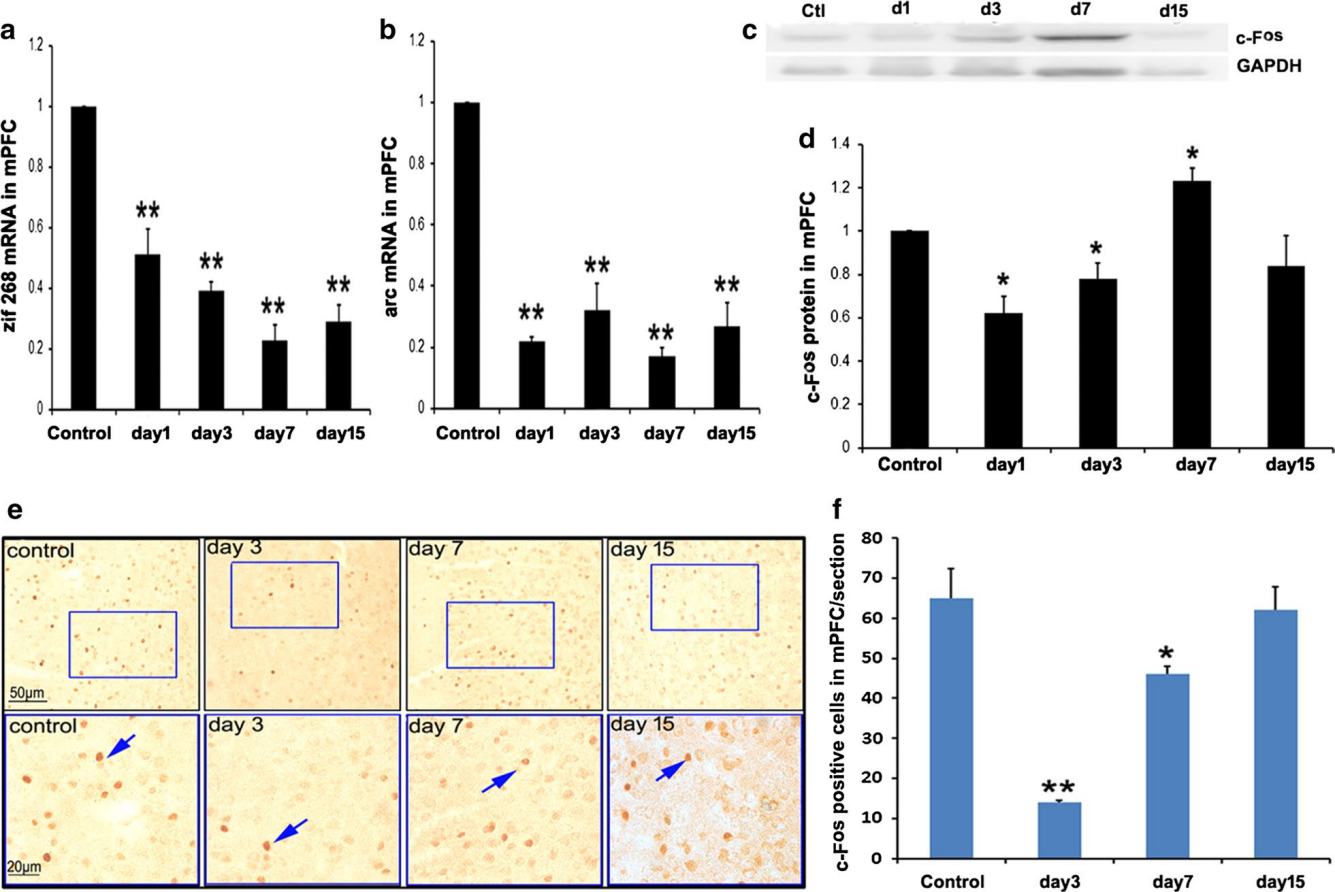
Response of mPFC to social defeat stress. **a** mRNA levels of zif268 in mPFC tissues from intruder mice decreased at days 1, 3, 7 and 15 after stimulation compared with the control mice, one way ANOVA with Tukey’s test was used, F = 99.345, **p < 0.01 compared with the control, n = 8. **b** mRNA levels of arc in mPFC tissues from intruder mice decreased at days 1, 3, 7 and 15 after stimulation compared with the control mice, one way ANOVA with Tukey’s test was used, F = 43.18, **p < 0.01 compared with the control, n = 8. **c** Representative Western blot image of c-Fos. **d** Statistical analysis of c-Fos expression, one way ANOVA with Tukey’s test was used, F = 11.4, *p < 0.05, compared with the control, n = 8. **e** The representative images of c-Fos staining in mPFC. Squares in the upper images indicate areas that are shown magnified in the bottom images. Blue arrows indicate c-fos-positive cells. **f** Cell counts in the mPFC, one way ANOVA with Tukey’s test was used, F = 23.965, *p < 0.05, **p < 0.01 compared with the control, n = 4

Levels of zif268 and arc mRNA were analyzed by Q-RT-PCR. As shown in Fig. 4a (F = 99.345), b (F = 43.18), the levels of these genes decreased on days 1, 3, 7 and 15 after stimulation. The expression of c-Fos protein decreased at days 1 and 3 and increased at day 7 after stimulation (Fig. 4c, d, F = 11.4). c-Fos positive cells were labeled and counted as described in "Methods" section. As shown in Fig. 4e, f (F = 23.965), the number of c-Fos-positive cells in intruder mice decreased significantly at days 3 and 7 after stimulation compared with those in the control mice, and then increased to the same levels as those in the control mice at day 15 after stimulation.

### Effects of Mus microinjection to the BLA on social behavior

**Fig. 5:**
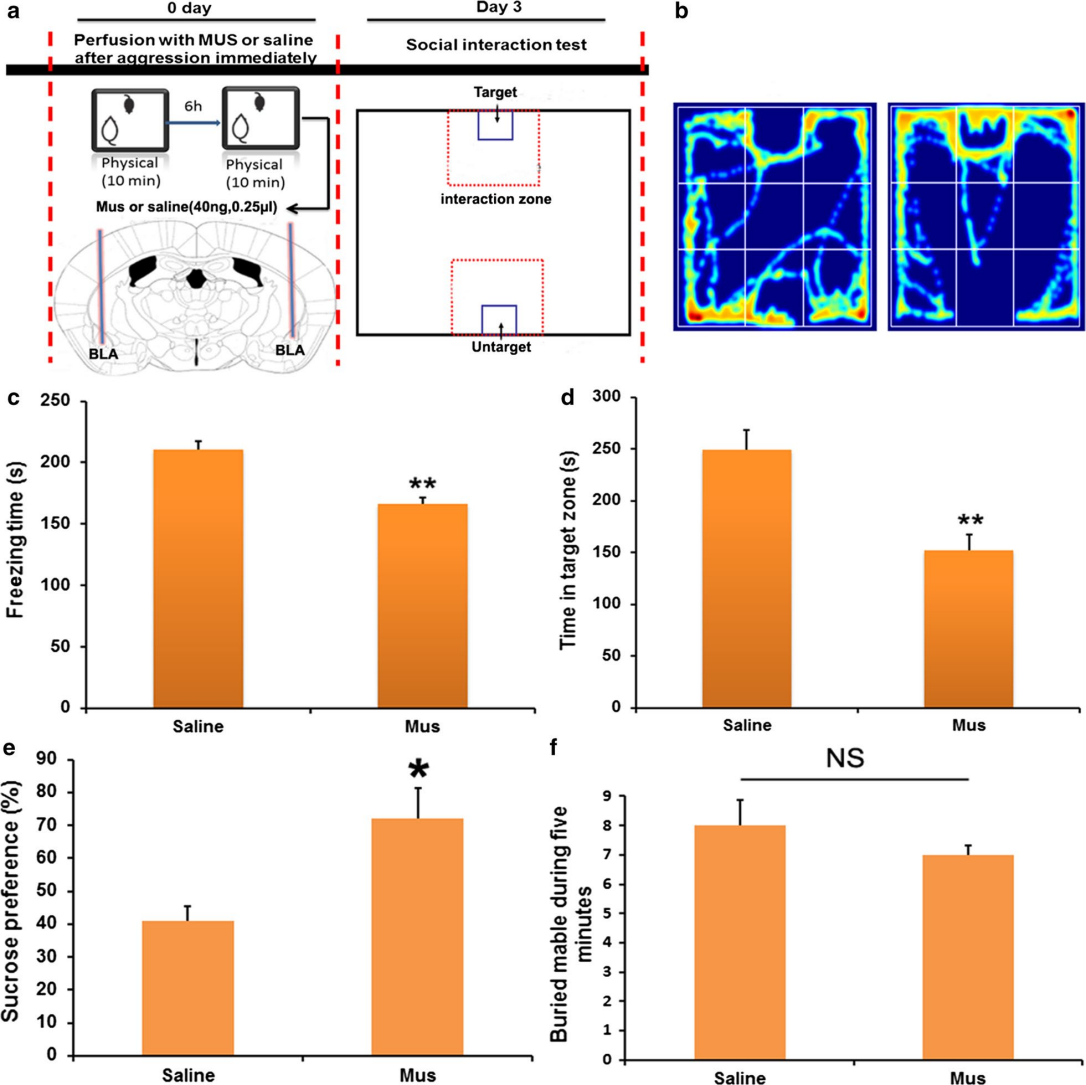
Effects of Mus microinjection to the BLA on social defeat stress. **a** Diagram of experimental flow. **b** Representative images of the moving track during social interaction. **c** Freezing time of intruder mice during a 5-min open-field test at day 3 after treatment. **d** Time spent in the social interaction zone at day 3 after treatment, compared with saline injection. **e** Sucrose reference at day 3 after treatment. **f** Marble burying test at day 3 after treatment. Independent student *t* test was used, *p < 0.05, **p < 0.01 compared with the saline-group, n = 8

Mus, as GABA_A_ receptor agonist were microinjected to BLA immediately after social aggression. Interestingly, on day 3 after microinjection with Mus or saline, the freezing time of the mice in the Mus-group was lower than that of the mice in the saline-group (Fig. 5c, p < 0.01). Also, the mice in the Mus-group spent more time on the interaction zone than the mice in the saline-group (Fig. 5b, d, p < 0.05). To identify whether social defeat accompanied depression or anxiety, sucrose preference and marble burying test were performed before test of social interaction. The results showed Mus injection increased sucrose preference (Fig. 5e, p < 0.05), but no significant effects on marble burying (Fig. 5f, p > 0.05), which suggested social defeat induced by acute social aggression accompanied depression-like behavior, but not anxiety.

### Effects of Mus microinjection to the BLA on IEGs expression

**Fig. 6:**
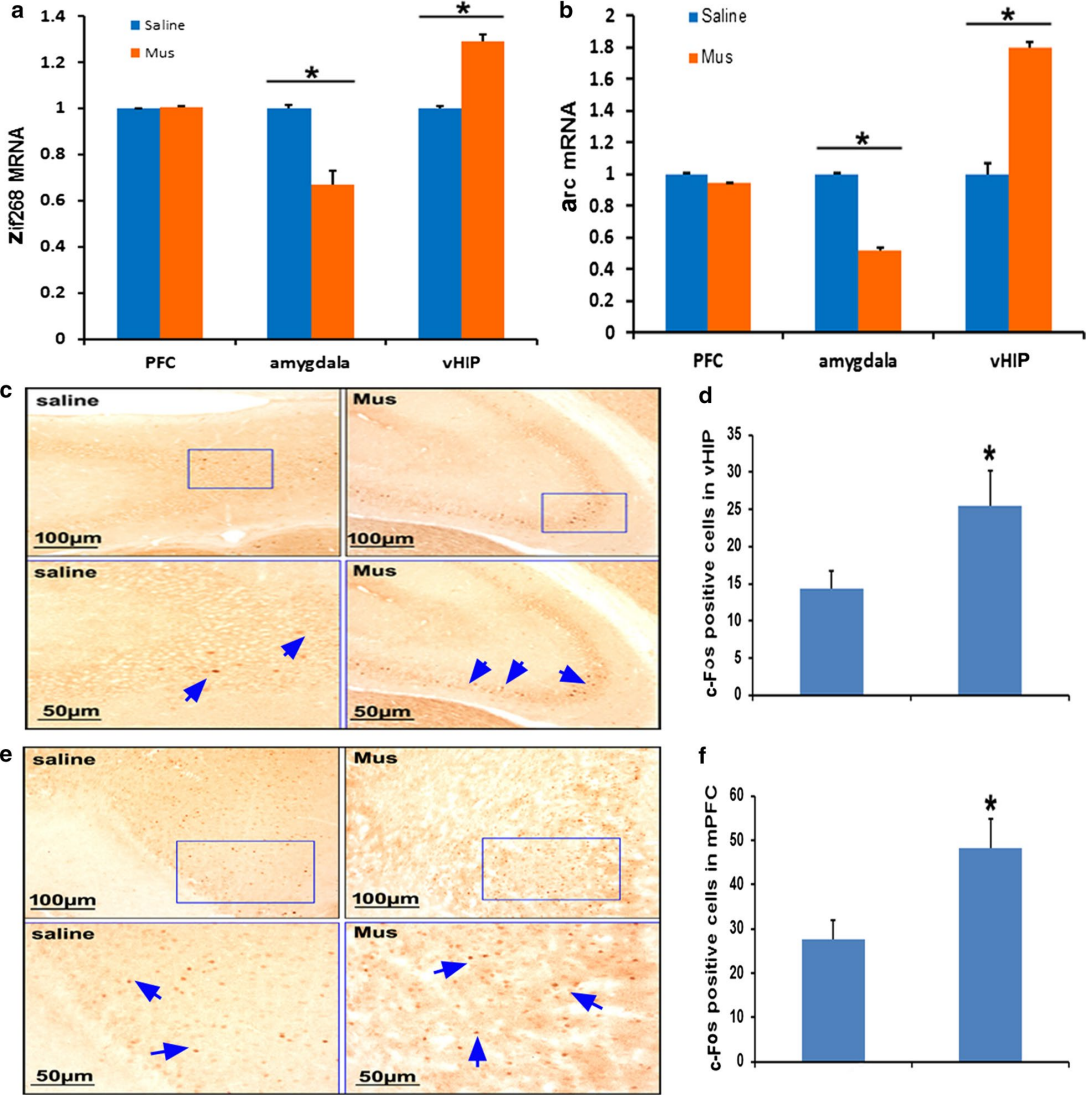
Effects of Mus microinjection to the BLA on IEGs expression. **a** zif268 mRNA expression levels in the BLA, vHIP and mPFC. **b** arc mRNA expression in the BLA, vHIP and mPFC. **c** Representative images of c-Fos in vHIP. **d** Numbers of c-Fos-positive cell in vHIP were calculated and statistically analyzed. **e** Representative images of c-Fos in mPFC. **f** Numbers of c-fos-positive cell in mPFC were calculated and statistically analyzed. Independent student *t* test was used, *p < 0.05 compared with the saline-group, n = 4

Mus were microinjected to BLA immediately after social aggression. At day 3 after social aggression, animals were sacrificed at 2 h after social interaction test. We quantified the expression levels of zif268 and arc in the BLA, vHIP and mPFC. As shown in Fig. 6a, b, the expression levels of zif268 and arc significantly decreased in the BLA and increased in the vHIP of the Mus-group compared with those in the saline-group (p < 0.05). In mPFC, no difference was observed between Mus-treated group and saline-treated group. c-Fos immunochemistry was performed to evaluated neural activity following social interaction test. In vHIP, numbers of c-Fos-positive cells increased in Mus-treated group compared with the saline-treated group (Fig. 6c, d, p < 0.05). In mPFC, numbers of c-Fos-positive cell also increased in Mus-treated group compared with the saline-treated group (Fig. 6e, f, p < 0.05).

## Discussion

We found that social defeat induced by acute aggression is characterized by social fear and avoidance, and is accompanied by a depression-like behavior. Behavioral test revealed that social fear was severe on days 1 and 3 and gradually diminished on days 7 and 15 after aggression. On days 1 and 3 after aggression, the expression of IEGs (including zif268, arc, and c-Fos) increased in the BLA but decreased in the vHIP and mPFC. On days 7 and 15 after aggression, the expression of IEGs decreased in the BLA but increased in the vHIP. Thus, BLA played an important role in establishing social defeat. Equal volumes of Mus and saline were microinjected into the bilateral BLA immediately after aggression to verify this hypothesis. On day 3 after aggression, social fear and freezing behavior were more significantly ameliorated by Mus treatment than by saline treatment. In addition, Mus treatment reduced the expression of IEGs in the BLA but increased that in the vHIP. The amygdala is critical in mediating fear- and anxiety-related behavior and is the key site for the acquisition and storage of fear memory. The plasticity of the sensory inputs from the thalamus and the cortical areas to the projection neurons in the BLA is the core mechanism underlying Pavlovian fear conditioning [23–25]. The associations between conditioned and unconditioned stimuli are formed through this mechanism. According to the neural circuit model of the amygdala function, fear output is generated by the associative information conveyed from the BLA by internuclear excitatory projections from the basal and basomedial nuclei to the medial region of the central amygdala. This passive information transfer model is currently being challenged because growing evidence indicates that the BLA and central amygdala can independently mediate parallel or additional associative functions under aversive conditioning [26, 27]. Another emerging evidence is that fear acquisition is controlled by the plasticity of the inhibitory synapses and the interneurons in the amygdala [28]. On the one hand, regulating the inhibition in the BLA and the specific inhibitory synapses on principal neurons in the BLA is associated with the behavioral suppression of fear following the extinction of learning. On the other hand, the activity of local GABAergic neurons is regulated by the plasticity of their excitatory inputs in the BLA and the central lateral amygdala [29]. A recent study revealed that specific local interneurons in the BLA were differentially recruited during conditioned and un-conditioned stimulation associations, thereby mediating the inhibition and disinhibition of distinct subcellular domains for controlling fear learning. Therefore, multiple plastic systems, particularly those integrating GABAergic neurons, can collectively encode aspects of the stimulus associations in the amygdala. The local interneurons in the BLA are heterogeneous interneurons displaying different morphological, electrophysiological, and neurochemical characteristics. Parvalbumin positive interneurons constitute approximately 40% of these interneurons and exert robust peri-somatic inhibitory effects on the projection neurons [30, 31]. We found that the ratio of c-Fos to parvalbumin double labeled cells in the BLA was approximately 20% of the total c-Fos immunopositive cells on day 3 after social aggression (Additional file 1: Figure S1). These findings suggested that the interneurons in BLA are involved in the regulation of social defeat.

The extinction of fear memory is one treatment method for post-traumatic stress disorder, because the establishment of a novel social interaction is accompanied by the extinction of previous social fear. In this study, the recovery due to social interaction was observed on day 15 after aggression, suggesting that the previous aversive stimulation was gradually diminished. Researchers have recently used a condition stimulus memory retrieval–extinction procedure to prevent fear recovery after extinction in rats and humans [32–35]. Consolidation refers to the process wherein memories are retained after their retrieval and destabilization. Destabilization refers to the return of a memory to a labile phase after memory retrieval. Researchers have inferred that memory consolidation is hampered when the expression of the conditioned responses is disrupted by post-retrieval neural pharmacological manipulations within a specific time interval (i.e., a “reconsolidation window” of up to 2 h post retrieval. This strategy effectively promoted the extinction of aversive memory, which aided in ameliorating the symptoms of mood disorders [36–38]. Our results suggested that aversive memory became extinct after 15 days without additional social defeat. Once the aversive information is forgotten, the learning ability for a novel stimulation is recovered. Another recent study found that autism is associated with genetic variation and the copy number deletion of P-Rex1, which encodes the phosphatidylinositol-3,4,5-trisphosphate-dependent Rac exchange factor1. The genetic deletion or knockdown of P-Rex1 in the CA1 region of the mouse hippocampus produced autism-like behaviors, such as impaired social interactions, reversal learning deficits in the water maze, and extinction-resistant memory of fear conditioning [39, 40]. These behavioral changes were possibly due to impaired active forgetting or, in a general sense, the failure to update memories. Context recognition involves hippocampal function. The inhibition of the vHIP induced by social aggression disturbs the recognition of novel context and social objects at an early stage (on days 1 and 3 after social defeat). The hippocampal response to novel stimulation resulted in the recovery of the learning ability at a later stage (on days 7 and 15 after social defeat).

The mPFC is associated with motivation as the center of emotion integration. A previous study posited that the activation of mPFC diminished social defeat [41]. Deep brain stimulation in the mPFC ameliorated the symptoms of patients suffering from severe depression [42]. The social avoidance of the intruder mice reduced the expression of IEGs in the mPFC for novel contexts and social objects at an early stage of social defeat. The social interaction of the control mice led to the high expression of IEGs in the mPFC for novel social objects. The inhibition of the BLA by Mus treatment increased the social interaction and exploration time for novel contexts, suggesting that BLA establishes and consolidates social information. Stress recovery was aided by inhibiting the function of BLA after an aversive stimulation at an early stage. Disturbances at critical times affect the efficacy of the consolidation or extinction of the aversive stimulation. Consolidation and extinction are based on synaptic plasticity and new protein synthesis, both of which are dependent on the duration of the neural activity. Therefore, the time window of a treatment is important. Salinas reported that training-related IGF-II-dependent memory enhancement is restricted to a temporal window of less than a day. However, the enhancing effect re-emerges at a later time if IGF-II is combined with memory retrieval, which reactivates the memory and induces reconsolidation. The IGF-II effect following retrieval is also temporally limited and restricted to a temporal window that overlaps with the reconsolidation-sensitive period of inhibition avoidance [43]. For the retrieval-induced memory fragility and the IGF-II-dependent enhancement, both of them require new protein synthesis,but in different brain regions, such as the amygdala in the former and the hippocampus in the latter. Therefore, social fear memory was in a sensitive period during the first 3 days after aggression. When activated, this memory could either be significantly weakened or enhanced. These findings suggested that reconsolidation is a lingering consolidation process [44, 45].

Although the methodology was limited, our data revealed the duration of social defeat from consolidation to extinction after an acute social aggression. In the future, we will explore the relationship between the responses of the BLA and the vHIP toward social defeat, particularly the functions of the neural circuit between the BLA and the vHIP in recognizing social contexts and objects. We aim to use extinction training and noninvasive treatment to alleviate social defeat and explore the mechanisms underlying the temporal windows of consolidation and extinction.

## Conclusion

Acute social defeat was induced by using a social aggressive attack according to the intruder–resident paradigm. The social behavior of the intruder mice toward novel contexts and objects was measured on days 1, 3, 7, and 15 after aggression. The expression of IEGs in the BLA, vHIP, and mPFC was analyzed at 2 h after behavioral test. The results indicated that social fear and avoidance were significant on days 1–3 after aggression and were accompanied by a high expression of IEGs in the BLA and a low expression of IEGs in the vHIP and mPFC. On days 7–15 after aggression, the social interaction gradually increased and was accompanied by a low expression of IEGs in the BLA and a high expression of IEGs in the vHIP. Inhibiting the function of BLA after aggression ameliorated social defeat, which suggested that BLA consolidates social fear. This study posited that social defeat can be treated by suppressing the consolidation or prompting the extinction of aversive stimulations.

## Additional file

**Additional file 1: Figure S1.** To double-label BLA with c-fos and parvalbumin, BLA sections were stained with parvalbumin and developed with DAB. c-Fos was labeled and developed with DAB-nickel ammonium sulfate. Representative images of c-Fos and parvalbumin double labeling, blue arrows indicate the double labelling cells; black arrows indicate the single labelling cells.

## References

[1] Kessler RC, Akiskal HS, Ames M, Birnbaum H, Greenberg P, Hirschfeld RM (2006). Prevalence and effects of mood disorders on work performance in a nationally representative sample of U.S. workers. Am J Psychiatry.

[2] Han B, Compton WM, Blanco C, Colpe LJ (2017). Prevalence, treatment, and unmet treatment needs of US adults with mental health and substance use disorders. Health Aff.

[3] Toyoda A (2017). Social defeat models in animal science: what we have learned from rodent models. Anim Sci J.

[4] Blanchard DC, Griebel G, Blanchard RJ (2003). Conditioning and residual emotionality effects of predator stimuli: some reflections on stress and emotion. Prog Neuropsychopharmacol Biol Psychiatry.

[5] Treit D, Pinel JP, Fibiger HC (1981). Conditioned defensive burying: a new paradigm for the study of anxiolytic agents. Pharmacol Biochem Behav.

[6] Dabrowska J, Hazra R, Ahern TH, Guo JD, McDonald AJ, Mascagni F (2011). Neuroanatomical evidence for reciprocal regulation of the corticotrophin-releasing factor and oxytocin systems in the hypothalamus and the bed nucleus of the stria terminalis of the rat: implications for balancing stress and affect. Psychoneuroendocrinology.

[7] Cooper MA, McIntyre KE, Huhman KL (2008). Activation of 5-HT1A autoreceptors in the dorsal raphe nucleus reduces the behavioral consequences of social defeat. Psychoneuroendocrinology.

[8] Markham CM, Taylor SL, Huhman KL (2010). Role of amygdala and hippocampus in the neural circuit subserving conditioned defeat in Syrian hamsters. Learn Mem.

[9] Jasnow AM, Shi C, Israel JE, Davis M, Huhman KL (2005). Memory of social defeat is facilitated by cAMP response element-binding protein overexpression in the amygdala. Behav Neurosci.

[10] Taylor SL, Stanek LM, Ressler KJ, Huhman KL (2011). Differential brain-derived neurotrophic factor expression in limbic brain regions following social defeat or territorial aggression. Behav Neurosci.

[11] Bader LR, Carboni JD, Burleson CA, Cooper MA (2014). 5-HT1A receptor activation reduces fear-related behavior following social defeat in Syrian hamsters. Pharmacol Biochem Behav.

[12] Klavir O, Prigge M, Sarel A, Paz R, Yizhar O (2017). Manipulating fear associations via optogenetic modulation of amygdala inputs to prefrontal cortex. Nat Neurosci.

[13] Parthoens J, Verhaeghe J, Stroobants S, Staelens S (2014). Deep brain stimulation of the prelimbic medial prefrontal cortex: quantification of the effect on glucose metabolism in the rat brain using [(18) F]FDG microPET. Mol Imaging Biol.

[14] Czerniawski J, Ree F, Chia C, Ramamoorthi K, Kumata Y, Otto TA (2011). The importance of having Arc: expression of the immediate-early gene Arc is required for hippocampus-dependent fear conditioning and blocked by NMDA receptor antagonism. J Neurosci.

[15] McDonald AJ (1998). Cortical pathways to the mammalian amygdala. Prog Neurobiol.

[16] Green MR, Barnes B, McCormick CM (2013). Social instability stress in adolescence increases anxiety and reduces social interactions in adulthood in male Long-Evans rats. Dev Psychobiol.

[17] Razzoli M, Carboni L, Andreoli M, Ballottari A, Arban R (2011). Different susceptibility to social defeat stress of BalbC and C57BL6/J mice. Behav Brain Res.

[18] Venzala E, Garcia-Garcia AL, Elizalde N, Tordera RM (2013). Social vs. environmental stress models of depression from a behavioural and neurochemical approach. Eur Neuropsychopharmacol.

[19] Beiderbeck DI, Neumann ID, Veenema AH (2007). Differences in intermale aggression are accompanied by opposite vasopressin release patterns within the septum in rats bred for low and high anxiety. Eur J Neurosci.

[20] Filiano AJ, Martens LH, Young AH, Warmus BA, Zhou P, Diaz-Ramirez G (2013). Dissociation of frontotemporal dementia-related deficits and neuroinflammation in progranulin haploinsufficient mice. J Neurosci.

[21] Iniguez SD, Riggs LM, Nieto SJ, Dayrit G, Zamora NN, Shawhan KL (2014). Social defeat stress induces a depression-like phenotype in adolescent male c57BL/6 mice. Stress.

[22] Mouri A, Ukai M, Uchida M, Hasegawa S, Taniguchi M, Ito T (2018). Juvenile social defeat stress exposure persistently impairs social behaviors and neurogenesis. Neuropharmacology.

[23] An B, Kim J, Park K, Lee S (2017). Amount of fear extinction changes its underlying mechanisms. Elife.

[24] Bocchio M, Nabavi S, Capogna M (2017). Synaptic plasticity, engrams, and network oscillations in amygdala circuits for storage and retrieval of emotional memories. Neuron.

[25] Moscarello JM, LeDoux JE (2013). Active avoidance learning requires prefrontal suppression of amygdala-mediated defensive reactions. J Neurosci.

[26] Jimenez SA, Maren S (2009). Nuclear disconnection within the amygdala reveals a direct pathway to fear. Learn Mem.

[27] Trouche S, Sasaki JM, Tu T, Reijmers LG (2013). Fear extinction causes target-specific remodeling of perisomatic inhibitory synapses. Neuron.

[28] Polepalli JS, Wu H, Goswami D, Halpern CH, Sudhof TC, Malenka RC (2017). Modulation of excitation on parvalbumin interneurons by neuroligin-3 regulates the hippocampal network. Nat Neurosci.

[29] McDonald AJ, Betette RL (2001). Parvalbumin-containing neurons in the rat basolateral amygdala: morphology and co-localization of Calbindin-D (28 k). Neuroscience.

[30] Lucas EK, Jegarl AM, Morishita H, Clem RL (2016). Multimodal and site-specific plasticity of amygdala parvalbumin interneurons after fear learning. Neuron.

[31] Wolff SB, Grundemann J, Tovote P, Krabbe S, Jacobson GA, Muller C (2014). Amygdala interneuron subtypes control fear learning through disinhibition. Nature.

[32] Monfils MH, Cowansage KK, Klann E, LeDoux JE (2009). Extinction-reconsolidation boundaries: key to persistent attenuation of fear memories. Science.

[33] Clem RL, Huganir RL (2010). Calcium-permeable AMPA receptor dynamics mediate fear memory erasure. Science.

[34] Schiller D, Monfils MH, Raio CM, Johnson DC, Ledoux JE, Phelps EA (2010). Preventing the return of fear in humans using reconsolidation update mechanisms. Nature.

[35] Schiller D, Kanen JW, LeDoux JE, Monfils MH, Phelps EA (2013). Extinction during reconsolidation of threat memory diminishes prefrontal cortex involvement. Proc Natl Acad Sci USA.

[36] Milton AL, Everitt BJ (2010). The psychological and neurochemical mechanisms of drug memory reconsolidation: implications for the treatment of addiction. Eur J Neurosci.

[37] Nader K, Schafe GE, Le Doux JE (2000). Fear memories require protein synthesis in the amygdala for reconsolidation after retrieval. Nature.

[38] Alberini CM (2005). Mechanisms of memory stabilization: are consolidation and reconsolidation similar or distinct processes?. Trends Neurosci.

[39] Shuai Y, Lu B, Hu Y, Wang L, Sun K, Zhong Y (2010). Forgetting is regulated through Rac activity in Drosophila. Cell.

[40] Davis RL, Zhong Y (2017). The biology of forgetting—a perspective. Neuron.

[41] Chaudhury D, Walsh JJ, Friedman AK, Juarez B, Ku SM, Koo JW (2013). Rapid regulation of depression-related behaviours by control of midbrain dopamine neurons. Nature.

[42] Accolla EA, Aust S, Merkl A, Schneider GH, Kuhn AA, Bajbouj M (2016). Deep brain stimulation of the posterior gyrus rectus region for treatment resistant depression. J Affect Disord.

[43] Chen DY, Stern SA, Garcia-Osta A, Saunier-Rebori B, Pollonini G, Bambah-Mukku D (2011). A critical role for IGF-II in memory consolidation and enhancement. Nature.

[44] Alberini CM, Milekic MH, Tronel S (2006). Mechanisms of memory stabilization and de-stabilization. Cell Mol Life Sci CMLS.

[45] Taubenfeld SM, Milekic MH, Monti B, Alberini CM (2001). The consolidation of new but not reactivated memory requires hippocampal C/EBPbeta. Nat Neurosci.

