# Supplementary figures and images for: Interaction of basolateral amygdala, ventral hippocampus and medial prefrontal cortex regulates the consolidation and extinction of social fear
Source: Behav Brain Funct. 2018 Mar 19;14:7. doi: 10.1186/s12993-018-0139-6 (PMC5858134; doi:10.1186/s12993-018-0139-6)

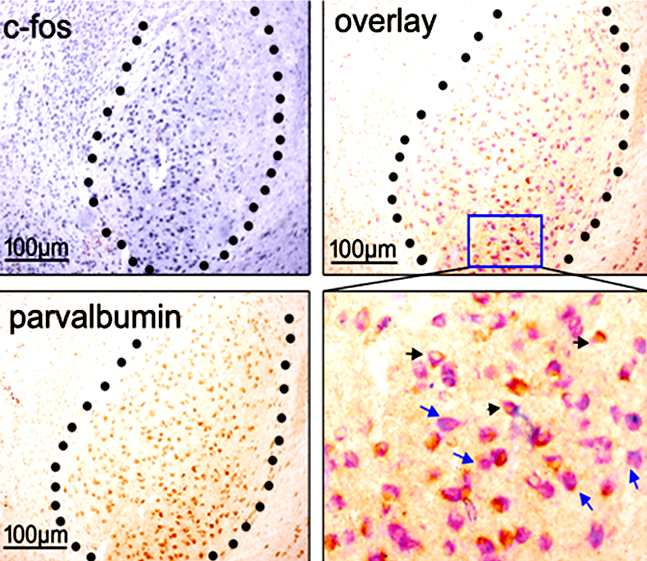

Supplement: Supplementary file 1 — Additional file 1: Figure S1. To double-label BLA with c-fos and parvalbumin, BLA sections were stained with parvalbumin and developed with DAB. c-Fos was labeled and developed with DAB-nickel ammonium sulfate. Representative images of c-Fos and parvalbumin double labeling, blue arrows indicate the double labelling cells; black arrows indicate the single labelling cells. [file 12993_2018_139_MOESM1_ESM.tif]
